# The association between stress hyperglycemia and poor outcome in critically ill children is modulated by hyperlactatemia

**DOI:** 10.3389/fendo.2025.1518746

**Published:** 2025-06-18

**Authors:** Wenjun Liu, Milan Dong, Jing Li, Guoying Zhang, Ju Chen, Jianyu Jiang, Ling Duan, Daoxue Xiong, Bo Huang, Yingbo Zou, Fuyan Liu, Hongmin Fu, Kai Yu

**Affiliations:** ^1^ Department of Critical Care Medicine, Children’s Hospital of Chongqing Medical University, National Clinical Research Center for Child Health and Disorders, Ministry of Education Key Laboratory of Child Development and Disorders, Chongqing Key Laboratory of Pediatric Metabolism and Inflammatory Diseases, Chongqing, China; ^2^ Department of Pediatrics, Women and Children’s Hospital of Chongqing Medical University, Chongqing Health Center for Women and Children, Chongqing, China; ^3^ Department of Pediatric Critical Care, Chengdu Women and Children’s Central Hospital, Chengdu, Sichuan, China; ^4^ Department of Pediatric Intensive Medicine, Chongqing University Three Gorges Hospital, Wanzhou, Chongqing, China; ^5^ Department of Pediatric Critical Care, The First People’s Hospital of Zunyi, Zunyi, Guizhou, China; ^6^ Department of Pediatric Critical Care, Kunming Children’s Hospital, Kunming, Yunnan, China

**Keywords:** critically ill, children, hyperlactatemia, stress hyperglycemia, outcome

## Abstract

**Background:**

The available evidence on tight glycemic control is conflicting, while the interaction between glucose and lactate in critically ill children remains unclear.

**Objective:**

To explore the potential role of hyperlactatemia (HL) in modulating the relationship between stress hyperglycemia (SHG) and poor outcomes, aiming to establish tailored glucose targets in critically ill children.

**Methods:**

This was a secondary analysis of a prospective observational cohort study conducted in five Pediatric Intensive Care Units (PICU) in southwestern China (ChiCTR2000030846). The interaction effect between glucose and lactate metrics concerning outcomes and subsequent subgroup regression analysis was conducted. SHG was defined as glucose > 150 mg/dL(8.3mmol/L) and HL as lactate > 2 mmol/L.

**Results:**

A cohort of 433 pediatric patients with 4885 arterial blood gas measurements were finally enrolled. 90 (20.8%) cases died within 28 days of PICU admission. Significant interaction effects between SHG and HL on outcomes were observed (p < 0.05). In the non-HL group, SHG was not an independent predictor of 28-day mortality (p = 0.656) and was not correlated with either 28-day ventilator-free days (p = 0.916) or 28-day ICU-free days (p = 0.914). In contrast, in the HL group, SHG was independently associated with 28-day mortality (OR 3.55, 95% CI 1.62~7.80, p = 0.002) and correlated with a reduction of 5.04 28-day ventilator-free days (p = 0.003) and 4.10 28-day ICU-free days (p = 0.004).

**Conclusions:**

HL potentially modulates the correlation between SHG and poor outcomes in pediatric critically ill patients. Combined SHG and HL are associated with poor outcomes, whereas SHG without HL is not.

## 1 Background

Stress hyperglycemia (SHG), with different diagnostic criteria occurring with prevalence rates ranging from 20% to 75% (1), is evident correlates with increased mortality and morbidity in extensive clinical settings, including emergency departments (2), cardiac care units (3), ICUs and so on (4, 5). Regardless of whether SHG acts as a causal factor or solely serves as a prognostic indicator, using insulin to achieve lower blood glucose targets appears to be a cost-effective strategy for improving patient prognosis. However, while studies such as the Leuven trial advocated tight glycemic control as beneficial for improving outcomes (6), other trials like NICE-SUGAR warned against it, citing its potential risk of increasing the 90-day mortality and the incidence of hypoglycemia (7). The conflicting findings leave the optimal approach for critically ill patients widely controversial, underscoring the necessity for a novel approach to uncover potential confounders within this context.

Lactate, also a robust prognostic marker, along with glucose, can exhibit increased levels in response to stress to fulfill heightened energy requirements (8, 9). These two crucial metabolites are intricately linked in carbohydrate metabolism, serving as precursors for each other and undergoing mutual conversion within the biological processes of glycolysis and gluconeogenesis (10, 11). It is recognized that during physiological stress, roughly 60% of blood glucose originates from lactate in certain circumstances (12). Given this notable interdependence, assessing the correlation between SHG and adverse outcomes while considering lactate levels simultaneously may be more appropriate. Several publications have indeed reported lactate as a modulator of the relationship between increased death risk and SHG (13–17). By accounting for lactate levels, the association between SHG and mortality appears to vanish, suggesting that blood glucose targets should be tailored depending on lactate levels in critically ill patients (13–15).

The current body of research primarily centers around the adult ICU cohorts (13–17). To our understanding, there is a conspicuous lack of studies addressing this issue in critically ill children. We put forth a hypothesis that hyperlactatemia (HL) could potentially modulate the association between SHG and unfavorable prognosis in critically ill pediatric cohorts, to contribute valuable insight into glycemic control strategy for this particular population.

## 2 Materials and methods

### 2.1 Study design and participants

This was a secondary analysis of a prospective observational cohort study (ChiCTR2000030846) (18). Patients were consecutively monitored in the five Pediatric Intensive Care Units (PICUs) in southwestern China from January to December 2020. To be eligible for inclusion, participants must: 1) receive vasoactive drug support for hypotension or ventilatory support for respiratory failure; 2) be between a corrected gestational age of 36 weeks and 16 years; 3) remain in the PICU for more than 24 hours. Exclusion criteria are: 1) a prior diagnosis of diabetes mellitus or inborn errors of metabolism; 2) readmission to the PICU; 3) use of glucocorticoids or total parenteral nutrition; 4) fewer than two arterial blood gas analyses daily during the first 72 hours of ICU admission. This secondary analysis was approved by the ethics committee of Children’s Hospital of Chongqing Medical University and was granted a waiver for informed consent (File No. 2022,125). Elaboration on the methods for original data collection, inclusion and exclusion criteria, insulin usage protocols, and quality control procedures can be referenced in our previous study (18).

### 2.2 Data collection

We collected baseline demographic data (age, gender, and nutritional status), primary reasons for ICU admission (urgent organ dysfunction or critical disease conditions necessitating ICU intervention), clinical treatments (such as insulin, glucose infusion rate (GIR), and other supportive therapies), and illness severity assessment (The Pediatric Logistic Organ Dysfunction score-2, PELOD-2). The nutritional status index was assessed using the Z score for weight/height (for individuals aged ≤5 years) or Body Mass Index (for those older than five years) for age (BMZ), calculated through the Epi-info software. The PELOD-2 score was determined based on the most extreme physiological parameters and laboratory findings observed within the first 24 hours of admission, reflecting the severity of the patient’s condition, with a higher score indicating a worse condition and poorer prognosis (19).

### 2.3 Monitoring of glucose and lactate, and definitions

Blood Glucose and lactate measurements were exclusively obtained from arterial blood gas analyses during the first 72 hours after PICU admission. We obtained the concentration of mean glucose (Glu_mean_), maximum glucose (Glu_max_) and calculated time-weighted mean glucose (Glu_twmean_) (14). For lactate, corresponding measurements were collected, including mean lactate (Lac_mean_), maximum lactate concentration (Lac_max_), and time-weighted mean lactate (Lac_twmean_). All blood gas samples were obtained through routine monitoring or measurements based on the clinical judgment of the attending physicians, with no additional blood gas analyses performed specifically for the purpose of the study. The blood gas analyzers in different research centers shared similar reference ranges and underwent regular testing as well as stringent laboratory quality control measures, including daily internal quality control and biannual external quality assessments.

A criterion of at least one episode of blood glucose concentration above 150 mg/dL (8.3 mmol/L) was established for defining SHG, as this threshold marked the onset of increased mortality risk and was recommended for initiating insulin therapy (5, 20). Meanwhile, cut-off blood glucose concentrations of 65 mg/dL (3.6 mmol/L) and 40 mg/dL (2.2 mmol/L) were adopted to define mild hypoglycemia (21) and severe hypoglycemia (7), respectively. HL was defined as a blood lactate concentration greater than 2 mmol/L, as this cut-off value was the threshold for septic shock definition and was highly correlated with an unfavorable prognosis (22).

### 2.4 Study outcomes

The primary outcome of interest was 28-day mortality. The secondary outcomes comprised 28-day ventilator-free days and 28-day ICU-free days. A value of 0 was recorded for patients who did not survive within the 28 days, while the recorded value for survivors was 28 days minus the corresponding number of days on the ventilator or in the ICU, with a smaller value indicating a poorer prognosis (23).

### 2.5 Statistical analysis

All statistical analyses were performed using SPSS (Version 23, IBM, Armonk, NY, USA). The Kolmogorov-Smirnov test was used to assess the normality of continuous variables. The results showed that these variables had non-normal distributions, therefore, presenting as medians with interquartile ranges (IQR) and compared by the Wilcoxon rank-sum test. Categorical variables were expressed as numbers and percentages, and comparisons were made using either the chi-square test or the Yates corrected chi-square test, as appropriate. The Cochran-Mantel-Haenszel test was used to examine the association between SHG and 28-day mortality across different HL states, and the Breslow-Day test was used to assess the homogeneity of odds ratios (OR) across different strata. Interaction effects were evaluated by testing the product terms of lactate and glucose metrics in multivariable regression models. In cases of significant interaction effects, further subgroup regression analyses stratified by HL would be conducted. Depending on the type of outcome variable, either a multivariable logistic regression or linear regression model was employed. ORs with 95% confidence intervals (CI) derived from logistic regression were reported. For linear regression, coefficients, standard errors, and t-values were reported. Predefined covariates in the regression models were chosen based on demographics (gender, age groups cut-off at 36 months), nutritional status (BMZ), factors potentially influencing glycometabolism (GIR and insulin), and severity of illness (PELOD-2 score). To ensure the robustness of the results while adhering to predictor variable constraints and mitigating overfitting, additional covariates were sequentially introduced and replaced one at a time in subsequent steps. Sensitivity analyses were also performed by exploring the interaction effects of SHG and HL on outcomes in patients without hypoglycemia. A p-value < 0.05 for a two-sided test was considered statistically significant, except for interaction analyses where a p-value < 0.10 was used (24).

## 3 Results

### 3.1 Cohort characteristics

**Figure 1:**
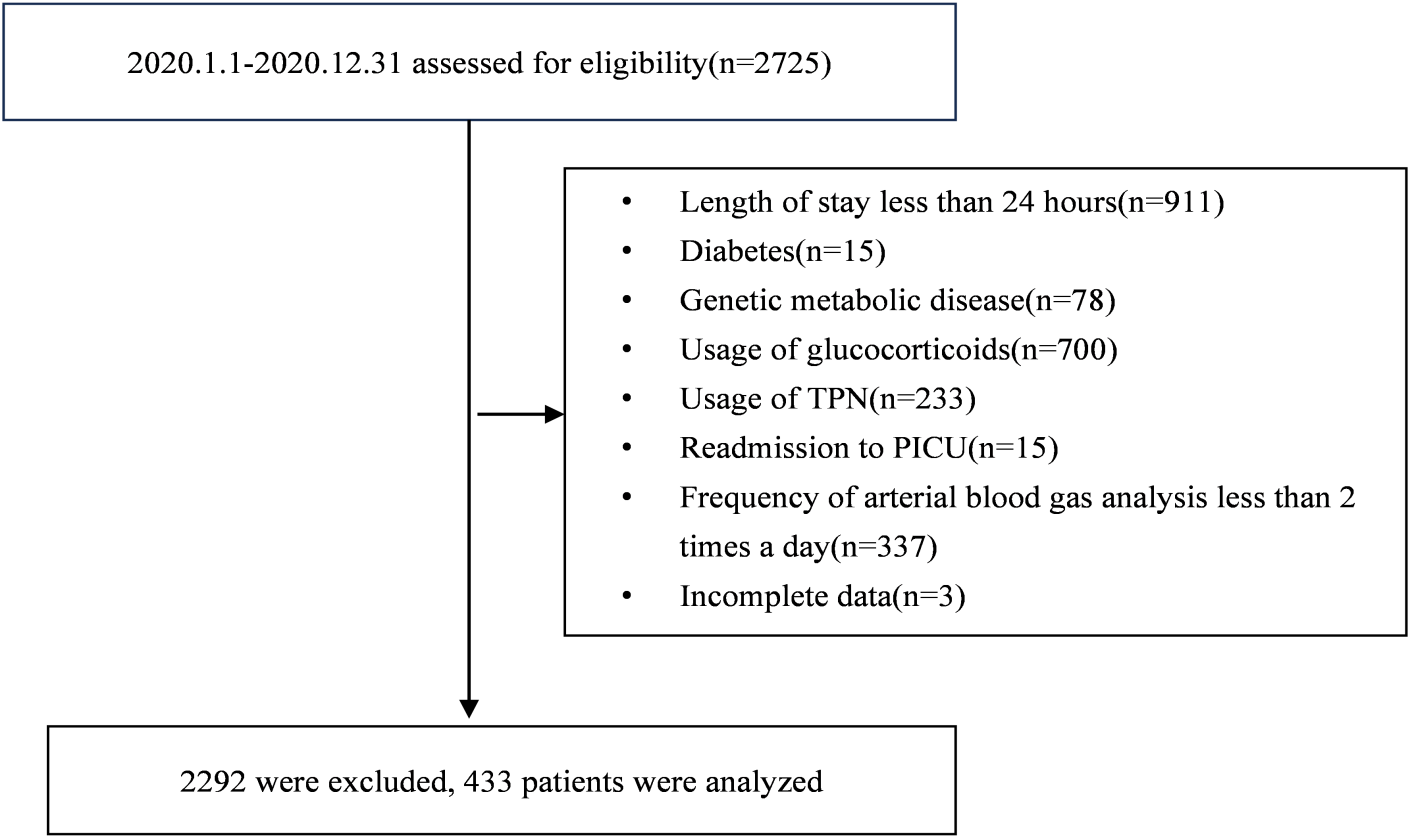
Flowchart illustrating participant selection for the study.

During the original study period, a total of 2,725 consecutive patients across the five participating units underwent stringent screening to determine their eligibility. Ultimately, a cohort of 433 pediatric patients was included (**Figure 1**). Among them, the median age was 16.0 (IQR: 4.3 ~ 71.5) months, 255 (58.9%) cases were males, and 90 (20.8%) died within 28 days after PICU admissions. The leading primary reason for ICU admission was respiratory diseases, accounting for 152 cases (35.1%). At the primary endpoint of the 28-day mortality assessment, participants were divided into survivors and non-survivors groups. The deceased cases were associated with elevated GIR, increased requirements of insulin, continuous renal replacement therapy, mechanical ventilation, vasoactive drugs, and higher PELOD-2 scores (p < 0.05).

Within the first 72 hours of PICU admission, 4885 simultaneous glucose and lactate measurements from arterial blood gas analyses were obtained, with a median frequency of 12 (IQR: 9~13) per patient. Compared with survivors, more cases of SHG (64.4% vs. 32.7%), mild hypoglycemia (34.3% vs. 21.6%), severe hypoglycemia (7.8% vs. 1.7%), and HL (71.1% vs. 36.2%) were detected in non-survivors (p<0.5) (**Table 1**).

**Table 1:** Characteristics of patient cohorts.

Variable	All subjects (n=433)	Survivors (n=343)	non-survivors (n=90)	*χ2/Z*	*p*
Age, month	16.0 (4.3~71.5)	15.0 (4.0~71.0)	19.0 (5.5~77.0)	0.817	0.414
Male**/**n (%)	255 (58.9)	200 (58.3)	55 (61.1)	0.231	0.631
BMZ/n (%)					
<-2	89 (20.5)	66 (19.2)	23 (25.6)	5.031	0.102
-2~2	316 (73.0)	251 (73.2)	65 (72.2)		
>2	28 (6.5)	26 (7.6)	2 (2.2)		
Primary reason for ICU admission					
Respiratory disease	152 (35.1)	123 (35.9)	29 (32.2)	0.875	0.350
Neurologic disorder	79 (18.2)	57 (16.6)	22 (24.4)		
Trauma	72 (16.6)	65 (19.0)	7 (7.8)		
Sepsis	53 (12.2)	38 (11.1)	15 (16.7)		
Cardiovascular disease	34 (7.9)	26 (7.6)	8 (8.9)		
Postoperative care	14 (3.2)	13 (3.8)	1 (1.1)		
Others	29 (6.7)	21 (6.1)	8 (8.9)		
GlR (mg/kg/min)	0.89 (0.49~1.50)	0.84 (0.47~1.45)	1.04 (0.54~1.97)	2.377	0.017^*^
Insulin/n (%)	18 (4.2)	7 (2.0)	11 (12.2)	16.082	<0.001^*^
Continuous renal replacement therapy/n (%)	42 (9.7)	21 (6.1)	21 (23.3)	24.110	<0.001^*^
Mechanical ventilation/n (%)	355 (82.0)	269 (78.4)	86 (95.6)	14.165	<0.001^*^
Vasoactive drug/n (%)	143 (33.0)	90 (26.2)	53 (58.9)	34.360	<0.001^*^
PELOD-2 score	6.0 (4.0~8.0)	5.0 (4.0~7.0)	8.0 (6.0~12.0)	7.508	<0.001^*^
Dysglycemia/n (%)					
SHG	170 (39.3)	112 (32.7)	58 (64.4)	30.216	<0.001^*^
Mild hypoglycemia	105 (24.2)	74 (21.6)	31 (34.4)	6.429	0.011^*^
Severe hypoglycemia	13 (3.0)	6 (1.7)	7 (7.8)	6.947	0.008^*^
HL/n **(%)**	188 (43.4)	124 (36.2)	64 (71.1)	35.468	<0.001^*^

BMZ, the “z” score for weight/height (for children ≤5 years old) and the BMI (for children >5 years old), overweight was classified as BMZ > +2SD, while wasting was BMZ < -2SD; GIR, glucose infused rate; PELOD-2, the Pediatric Logistic Organ Dysfunction score-2. SHG, stress hyperglycemia.

*, the difference between groups was significant.

### 3.2 Interaction effect of glucose and lactate metrics on the primary outcome without covariates

**Figure 2:**
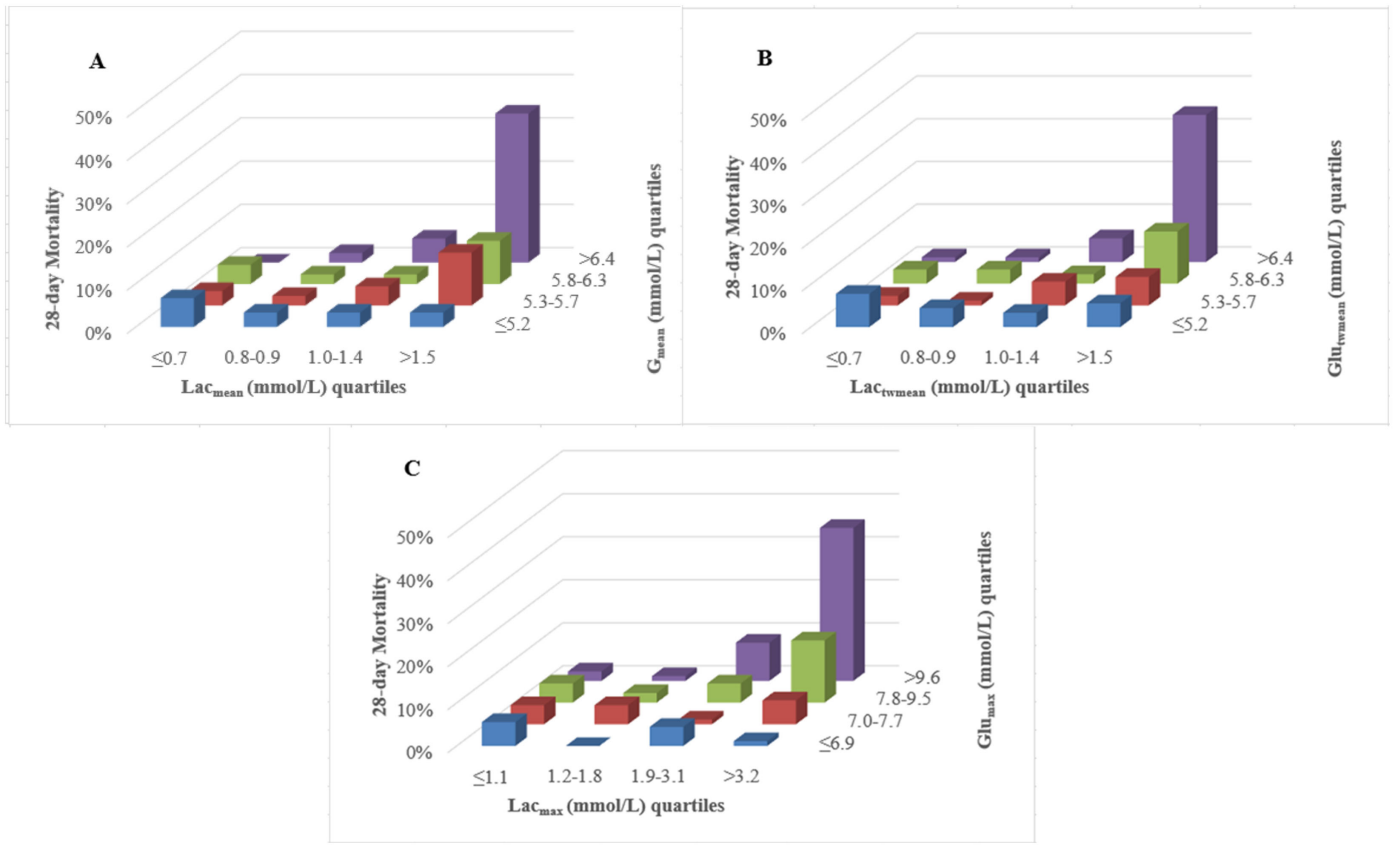
Three-dimensional column diagram of lactate and glucose quartiles related to 28-day mortality. **(A)** The combined highest Lac_mean_ and Glu_mean_ quartiles (upper-right column) indicated the highest 28-day mortality, whereas relatively low Lac_mean_ even with the highest Glu_mean_ didn’t correlate with high 28-day mortality (upper-left columns); **(B)** For the combination of Lac_twmean_ and Glu_twmean_, their highest quartiles (upper-right column) corresponded to peak 28-day mortality. However, lower Lac_twmean_ remained unrelated to high mortality, even with the highest Glu_twmean_ (upper-left columns); **(C)** The combined highest Lac_mean_ and Glu_max_ quartiles (upper-right column) corresponded to the highest 28-day mortality, while relatively low Lac_mean_, even at the highest Glu_mean_ quartiles, showed no association with elevated mortality (upper-left columns). Lac_mean_, mean lactate concentration; Lac_twmean_, time-weighted mean lactate; Lac_max_, maximum lactate concentration. Glu_mean_, mean glucose concentration; Glu_twmean_, time-weighted mean glucose; Glu_max_, maximum glucose concentration.

The three-dimensional column diagram illustrated 28-day mortality across combined quartiles of lactate and glucose metrics(**Figure 2**). **Figure 2A** revealed that the combined highest Lac_mean_ and Glu_mean_ quartiles (upper-right column) were associated with the highest 28-day mortality, whereas relatively low Lac_mean_ (Q1~Q2) even with the highest Glu_mean_ didn’t correlate with high 28-day mortality (upper-left columns); This pattern was consistently observed across quartiles of Lac_twmean_ and Glu_twmean_ (**Figure 2B**), as well as Lac_max_ and Glu_max_ (**Figure 2C**), concerning 28-day mortality.

**Figure 3:**
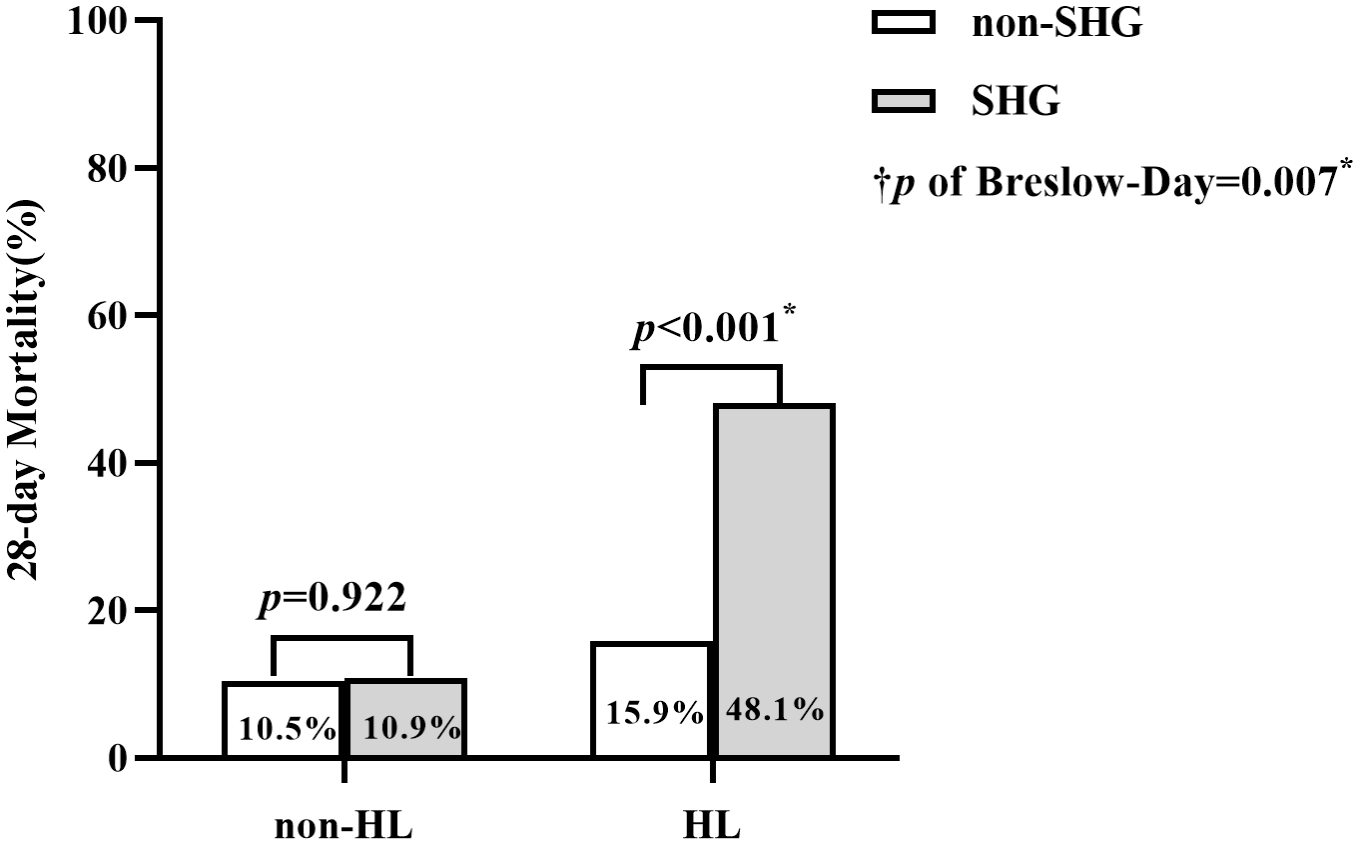
Comparison of 28-day Mortality between SHG and non-SHG group across HL and non-HL Subgroups. ^†^ *p*, the *p*-value from the Breslow-Day test (= 0.007) of Cochran-Mantel- Haenszel test, indicates a significant difference in the odds ratios (ORs) of the association between SHG and 28-day mortality between the HL and non-HL groups. *, the difference between groups was significant.

The Cochran’s and Mantel-Haenszel test was used to explore the relationship between SHG, HL, and 28-day mortality (**Figure 3**). In the non-HL group, SHG did not significantly affect PICU mortality (OR = 1.05, 95% CI 0.42~2.62, p = 0.922). Conversely, in the HL group, SHG was significantly associated with 28-day mortality (OR = 4.92, 95% CI 2.43~9.96, p < 0.001). The Test of Homogeneity of ORs revealed a significant difference in the ORs between the HL and non-HL groups (p of Breslow-Day = 0.007), indicating that the effect of SHG on mortality varied significantly with HL states.

### 3.3 Interaction effect of glucose and lactate metrics on the primary outcome with covariates

In the multivariate logistical regression analyses, noteworthy interaction effects were observed between Glu_mean_ and Lac_mean_, Glu_twmean_ and Lac_twmean_, Glu_max_ and Lac_max_, as well as SHG and HL concerning mortality, yielding p-values of interaction as 0.031, 0.020, 0.093 and 0.025, respectively. Stratified multivariate regression analyses revealed that in the subgroup without HL, quartiles of Glu_mean_, Glu_twmean_, and Glu_max_, as well as SHG, were not independent predictors of mortality (p = 0.377, 0.439, 0.572, and 0.656, respectively). Conversely, within the HL subgroup, all these glucose indices were independently associated with mortality: Glu_mean_ (OR 1.43, 95% CI 1.02~1.99, p = 0.036), Glu_twmean_ (OR 1.42, 95% CI 1.01~2.00, p = 0.042), Glu_max_ (OR 1.91, 95% CI 1.28~2.86, p = 0.002), and SHG (OR 3.55, 95% CI 1.62~7.80, p = 0.002) (**Table 2**). In addition, the interaction effects between other categorical covariates and HL/SHG were also checked, no significant interactions were found (p>0.1) (**Supplementary Table S1**).

**Table 2:** Multivariate logistic regression analysis of the association between glucose metrics and 28-day mortality in HL and Non-HL subgroups.

Glucose metrics	Non-HL (n=245)		HL (n=188)		†*p*-interaction
	OR 95%CI	*p*	OR 95%CI	*p*	
Glu_mean_	0.81 (0.51~1.29)	0.377	1.43 (1.02~1.99)	0.036^*^	0.031^*^
Glu_twmean_	0.83 (0.52~1.32)	0.439	1.42 (1.01~2.00)	0.042^*^	0.020^*^
Glu_max_	1.13 (0.74~1.71)	0.572	1.91 (1.28~2.86)	0.002^*^	0.093^*^
SHG	0.79 (0.28~2.26)	0.656	3.55 (1.62~7.80)	0.002^*^	0.025^*^

Glu_mean_, mean of glucose; Glu_twmean_, time weighted mean of glucose; Glu_max_, maximum of glucose. †*p*, the p value of interaction effect of glucose and lactate metrics (Glu_mean_ x Lac_mean_; Glu_twmean_ x Lac_twmean_; Glu_max_ x Lac_mean_; and SHG x HL, respectively); Except for the binary classification of SHG and HL, all other measurements were categorized into quartiles. †*p*-interaction <0.1 was considered significant [24]. *, the difference between groups was significant.

### 3.4 Interaction effect of SHG and HL on the secondary outcomes

Similarly, significant interaction effects between SHG and HL on 28-day ventilator-free days and 28-day ICU-free days were detected using multivariate linear regression, with p-values of 0.006 and 0.014, respectively. In the non-HL subgroup, no significant association between SHG and either 28-day ventilator-free days (p = 0.916) or 28-day ICU-free days (p = 0.914) was found. In contrast, within the HL subgroup, SHG was associated with a reduction of 5.04 days in 28-day ventilator-free days (p = 0.003) and 4.10 days in 28-day ICU-free days (p = 0.004), suggesting that HL may modulate the effect of SHG on these outcomes (**Table 3**).

**Table 3:** Multivariate linear regression analysis of the association between SHG and 28-day ventilator-free days, and ICU-free days, in HL and Non-HL subgroups.

Outcomes	Non-HL (n=245)				HL (n=188)				†p-interaction
	β	SE	t	p	β	SE	t	p	
28-day ventilator free days	0.12	1.21	0.10	0.916	-5.04	1.67	-3.04	0.003^*^	0.006^*^
28-day ICU free days	0.13	1.22	0.11	0.914	-4.10	1.42	-2.89	0.004^*^	0.014^*^

*β*, regression coefficient; SE, standard Error; †*p*, the interaction effect of SHG x HL in the unstratified multivariable linear regression. *, the difference between groups was significant.

### 3.5 Interaction effect of SHG and HL on the primary outcome with additional covariate adjustment

Additionally, to strengthen the observation that HL modulated the association between SHG and 28-day mortality, additional covariates were included. To adhere to predictor variable constraints and avoid overfitting, Mild hypoglycemia, severe hypoglycemia, continuous renal replacement therapy, mechanical ventilation, and vasoactive drugs were introduced and replaced sequentially. The interaction effect of SHG and HL remained robustly significant, with p-values of 0.019, 0.015, 0.022, 0.027, and 0.032, respectively. In the non-HL subgroup, SHG did not correlate with 28-day mortality (p > 0.05). In contrast, in the HL subgroup, SHG was consistently associated with mortality (p < 0.05) (**Table 4**).

**Table 4:** Multivariate logistic regression analysis of the association aetween SHG and 28-day mortality in HL and Non-HL subgroups with sequential covariate adjustment.

Additional covariate	Non-HL (n=245)		HL (n=188)		†*p* of interaction
	OR 95 CI%	*p*	OR 95 CI%	*p*	
Mild hypoglycemia	0.84 (0.29~2.40)	0.738	4.14 (1.81~9.48)	0.001^*^	0.019^*^
severe hypoglycemia	0.79 (0.28~2.27)	0.664	4.16 (1.81~9.60)	0.001^*^	0.015^*^
Continuous renal replacement therapy	0.68 (0.23~2.03)	0.492	3.39 (1.53~7.50)	0.003^*^	0.022^*^
Mechanical ventilation	0.78 (0.27~2.23)	0.780	3.44 (1.56~7.60)	0.002^*^	0.027^*^
Vasoactive drug	0.80 (0.28~2.29)	0.679	3.41 (1.54~7.53)	0.002^*^	0.032^*^

†*p*, the interaction effect of SHG x HL in the unstratified multivariable logistic regression. While maintaining baseline covariates constant, additional covariates were introduced and replaced sequentially.

### 3.6 Interaction effects of SHG and HL on 28-day mortality in patients without hypoglycemia

Recognizing the nature of the observational data, sensitivity analyses were further conducted. Patients with hypoglycemia at 3 thresholds: glucose <2.2, 3.3, and 3.6 mmol/L were sequentially excluded and formed 3 subgroups (excluded n=13, 59, 105; remaining n=420, 374, 328) (7, 21, 25). SHG×HL interactions on 28-day mortality and secondary outcomes of these 3 subgroups were retested. All subgroups showed consistent statical significance of interaction effects between HL, SHG and poor outcomes (p>0.1) (**Supplementary Tables S2**–**S7**).

## 4 Discussion

In this secondary analysis of a prospective, multicenter, observational study, the findings support our hypothesis that HL modulates the relationship between SHG and adverse outcomes in critically ill children. Subgroup analysis based on significant interaction effect revealed that, in participants without HL, there was no significant association between SHG and adverse outcomes. However, in patients with HL, SHG was significantly associated with an increased risk of 28-day mortality and a decreased duration of both ventilator-free and ICU-free days within 28 days. The findings suggest that prognostic models evaluating the effect of SHG in critically ill pediatric patients should also take lactate levels into account.

Before our work, a few observational studies reported that the association between SHG and adverse outcomes was modified by concomitant HL. A multicenter investigation examined static and time-weighted concurrent measurements of glucose and lactate from consecutive ICU patients and demonstrated that when lactate and glucose parameters were jointly considered, the independent correlation between glucose and mortality disappeared (14). Richards and colleagues also found that, in severely injured blunt trauma patients, there was no evidence for an association of glucose with mortality after adjusting for lactate (15). Another study observed that in a regression model of mortality, which included only mean lactate and mean glucose, the correlation between glucose and mortality vanished. The investigators also found a significant interaction effect between mean glucose and mean lactate concerning mortality (16). Similarly, we observed a sustained interaction effect between lactate and blood glucose metrics concerning mortality. Our results showed that isolated SHG was not associated with 28-day mortality (OR 0.79, 95% CI 0.28~2.26), but when combined with HL, the risk of SHG increased significantly (OR 3.55, 95% CI 1.62~7.80). Consistent with our findings, in nondiabetic septic adults, the OR for mortality risk was 3.96 (95% CI 2.01~7.79) when SHG and HL coexisted, whereas SHG without HL was not associated with increased mortality (OR 0.78, 95% CI 0.39~1.57) (13). Importantly, the available literature was all focusing on adult patients (13–16). Our study represents one of the few investigations highlighting that HL could modulate the relationship between SHG and adverse outcomes in severely ill children.

A combined assessment of glucose and lactate levels can reveal distinct glycometabolic states, where HL may serve as a critical biomarker of transitioning from adaptive to maladaptive glucose metabolism (26).During the early phase of critical illness, the body responds to increased secretion of catecholamines, glucagon, and corticosteroids, along with relative insulin resistance to promote glycogenolysis and gluconeogenesis (27–29). This physiological process helps to rapidly augment blood glucose concentrations to meet heightened energy demands (30, 31). In this stage, SHG is considered protective, resulting in greater plasticity and cellular resistance to ischemic and hypoxic insults (30). In this context, tight glycemic control at this stage may not be necessary and may be harmful. As critical illness progresses, oxidative phosphorylation fails to meet energy demands combined with SHG-induced mitochondrial impairment, prompting a shift to anaerobic glycolysis (32). At the same time, reduced hepatic lactate clearance, due to factors such as glucocorticoid resistance delaying gluconeogenesis, further contributes to a significant rise in lactate levels (33). The dynamic imbalance between glucose and lactate represents the collapse of energy metabolism homeostasis, with tricarboxylic acid cycle dysfunction as its core mechanism (34). Several studies have reported an association between SHG and concurrent HL (13, 17, 35, 36). Notably, a randomized multicenter trial involving post-cardiac surgery patients established that glucose peaks can induce corresponding lactate peaks a few hours later (37). However, a study found that the highest mortality was associated with the lowest glucose levels and the highest lactate levels, rather than with the highest levels of both (16). It can be explained that, in contrast to our more flexible insulin administration criteria, their patients adhered to a computerized, moderately strict glucose and potassium control protocol aimed at maintaining blood glucose levels below 8 mmol/L, which may have resulted in lower blood glucose levels and potentially masked the incidence of SHG.

Regarding clinical implications, tolerating SHG in the absence of concurrent HL is likely to offer prognostic benefits. SHG is widely reckoned as an evolutionary preserved adaptive response, as it may protect against cell death following ischemia by promoting anti-apoptotic pathways and cell survival pathways and favoring angiogenesis (30). On the other hand, persistent SHG can also be detrimental (38). The Society of Critical Care Medicine recommended initiating insulin therapy when blood glucose levels reach 150 mg/dL (20), and an updated guideline suggested treating persistent hyperglycemia using a threshold of 180 mg/dL (39). Although the optimal blood glucose target for ICU patients has not yet been determined, a range of 140 to 180 mg/dL is widely considered acceptable (38). Our finding offers valuable insights for clinicians’ bedside decision-making. When arterial blood gas analysis shows both glucose levels above 150 mg/dL and lactate levels above 2 mmol/L, this indicates a high-risk profile that requires prompt intervention. In these cases, increased monitoring or early insulin initiation may be warranted. This approach aligns with guidelines that encourage to seek explicit support tools to guide glycemic control (39). Notably, a recent study recommended moderate glycemic control (maintaining glucose levels between 8.3~10 mmol/L with insulin) for ICU patients who cannot eat and have arteriovenous catheters (40). Our research findings may contribute to bridging the gap in this range of glucose, where insulin use could be further tailored based on whether pediatric patients have concomitant HL. However, the evidence level of our study remains insufficient to warrant modifications to international and national guidelines at this time. The study has the following strengths. First, our study stands out as one of the few that sheds light on whether HL can alter the association between SHG and poor prognosis and provides a fresh perspective on blood glucose management in critically ill children. Second, the data used in the study were collected prospectively, which reduces the likelihood of bias. However, we also have important limitations. First, our blood glucose monitoring was intermittent rather than continuous, and only patients with more severe conditions underwent frequent blood gas testing. This may have overlooked some extreme values and introduced ascertainment bias, although it reflects the practical experience in most centers. Second, our sample size was relatively small due to the short duration of the original study (one year) and the stringent inclusion and exclusion criteria designed to control factors that could interfere with blood glucose levels. Despite this, the inclusion of multiple blood glucose metrics and consistent results across regression models strengthened our conclusion. Third, the sample size and the type of secondary analysis limited the number of covariates, such as inflammatory markers, lipid profiles, and comprehensive illness severity scores beyond PELOD-2, which may affect the relationships between HL, SHG, and prognosis. Future studies containing more centers and participants are needed to validate our findings. Fourth, the small sample size also limited the ability to create sufficiently large subgroups (e.g., by diagnosis such as heart failure, trauma, or severe sepsis), which may exhibit different metabolic responses influencing HL/SHG interactions. Larger studies with dedicated subgroup analyses are needed to explore potential differences. Last, because of the observational setting, we can only identify phenomena through data analysis and attempt to explain the mechanism.

## 5 Conclusion

HL potentially modulates the correlation between SHG and poor outcomes in critically ill pediatric patients. Combined SHG and HL are associated with poor outcomes, whereas isolated SHG is not. Profound research should focus on meticulously designed multicenter randomized controlled trials to determine whether allowing SHG alone and managing SHG only in conjunction with HL can improve prognostic outcomes.

## Data Availability

Some or all datasets generated during and/or analyzed during the current study are not publicly available but are available from the corresponding author on reasonable request.
