# Supplementary material for: The association between stress hyperglycemia and poor outcome in critically ill children is modulated by hyperlactatemia
Source: Front Endocrinol (Lausanne). 2025 Jun 18;16:1518746. doi: 10.3389/fendo.2025.1518746 (PMC12213459; doi:10.3389/fendo.2025.1518746)
Supplement: Supplementary file 1 [file Table1.docx]

**Supplementary Tables**

**Table S1: Interaction effects tests Between Additional Covariates, HL/SHG on 28-Day Mortality from Multivariable Logistic Regression Analysis**

|  | SHG, *p*-interaction | HL, *p*-interaction |
| --- | --- | --- |
| Male | 0.307 | 0.633 |
| Age group | 0.648 | 0.249 |
| BMZ | 0.380 | 0.188 |
| Insulin | -^a^ | 0.922 |
| Mild hypoglycemia | 0.352 | 0.976 |
| Severe hypoglycemia | 0.680 | 0.987 |
| Continuous renal replacement therapy | 0.215 | 0.980 |
| Mechanical ventilation | 0.715 | 0.476 |
| Vasoactive drug | 0.761 | 0.893 |

Interaction terms (covariate × SHG/HL) were tested one per model to avoid multicollinearity, with main effects retained as standard statistical practice. Baseline covariates matched those detailed in the statistical methods for consistent adjustment across analyses.

^a^,The interaction between SHG and insulin was not estimable because nearly all insulin users had SHG, leading to a lack of data variation needed to model their combined effect.

**Table S2. Multivariate Logistic Regression Analysis of the Association Between glucose metrics and 28-Day Mortality in HL and Non-HL Subgroups (Excluding cases with glucose<2.2mmolL)**

|  | **non-HL(n=244)** | |  | **HL(n=176)** | | †***p*-interaction** |
| --- | --- | --- | --- | --- | --- | --- |
|  | **OR 95%CI** | ***p*** |  | **OR 95%CI** | ***p*** |  |
| SHG without covariates | 1.07(0.43~2.67) | 0.892 |  | 6.42(2.89~14.26) | <0.001 | 0.004 |
| SHG with covariates | 0.80(0.28~2.27) | 0.664 |  | 4.72(1.95~11.41) | 0.001 | 0.011 |

†*p*, the p value of interaction effect of SHG x HL; The covariates included gender, age groups cut-off at 36 months, BMZ, GIR, insulin use, and PELOD-2 score.

**Table S3. Multivariate Linear Regression Analysis of the Association Between SHG and 28-Day Ventilator-Free Days, and ICU-Free Days, in HL and Non-HL Subgroups(Excluding cases with glucose<2.2mmolL)**

| **Outcomes** | **non-HL**  **(n=244)** | | | |  | **HL**  **(n=176)** | | | | †***p*-interaction** |
| --- | --- | --- | --- | --- | --- | --- | --- | --- | --- | --- |
|  | ***β*** | ***SE*** | ***t*** | ***p*** |  | ***β*** | ***SE*** | ***t*** | ***p*** |  |
| 28-day ventilator free days | 0.01 | 1.21 | 0.13 | 0.90 |  | -0.23 | 1.73 | -3.12 | 0.002 | 0.005 |
| 28-day ICU free days | 0.01 | 1.23 | 0.11 | 0.911 |  | -0.21 | 1.49 | -3.00 | 0.003 | 0.012 |

*β,* regression coefficient; SE, standard Error; †*p*, the p value of interaction effect of SHG x HL in the unstratified multivariable linear regression. The covariates included gender, age groups cut-off at 36 months, BMZ, GIR, insulin use, and PELOD-2 score.

**Table S4. Multivariate Logistic Regression Analysis of the Association Between glucose metrics and 28-Day Mortality in HL and Non-HL Subgroups (Excluding cases with glucose<3.3mmolL)**

|  | **non-HL(n=221)** | |  | **HL(n=153)** | | †***p*-interaction** |
| --- | --- | --- | --- | --- | --- | --- |
|  | **OR 95%CI** | ***p*** |  | **OR 95%CI** | ***p*** |  |
| SHG without covariates | 1.14(0.42~3.09) | 0.799 |  | 9.06(3.32~24.70) | <0.001 | 0.004 |
| SHG with covariates | 0.78(0.25~2.50) | 0.679 |  | 6.51(2.21~19.24) | 0.001 | 0.009 |

**Table S5. Multivariate Linear Regression Analysis of the Association Between SHG and 28-Day Ventilator-Free Days, and ICU-Free Days, in HL and Non-HL Subgroups(Excluding cases with glucose<3.3mmolL)**

| **Outcomes** | **non-HL**  **(n=221)** | | | |  | **HL**  **(n=153)** | | | | †***p*-interaction** |
| --- | --- | --- | --- | --- | --- | --- | --- | --- | --- | --- |
|  | ***β*** | ***SE*** | ***t*** | ***p*** |  | ***β*** | ***SE*** | ***t*** | ***p*** |  |
| 28-day ventilator free days | 0.01 | 1.26 | 0.12 | 0.907 |  | -0.23 | 1.84 | -3.06 | 0.003 | 0.004 |
| 28-day ICU free days | 0.02 | 1.29 | 0.27 | 0.786 |  | -0.21 | 1.56 | -2.78 | 0.006 | 0.009 |

**Table S6. Multivariate Logistic Regression Analysis of the Association Between glucose metrics and 28-Day Mortality in HL and Non-HL Subgroups (Excluding cases with glucose<3.6mmolL)**

|  | **non-HL(n=191)** | |  | **HL(n=137)** | | †***p*-interaction** |
| --- | --- | --- | --- | --- | --- | --- |
|  | **OR 95%CI** | ***p*** |  | **OR 95%CI** | ***p*** |  |
| SHG without covariates | 1.61(0.56~4.61) | 0.374 |  | 7.56(2.73~20.90) | <0.001 | 0.038 |
| SHG with covariates | 1.05(0.30~3.71) | 0.938 |  | 4.76(1.56~14.51) | 0.006 | 0.084 |

**Table S7. Multivariate Linear Regression Analysis of the Association Between SHG and 28-Day Ventilator-Free Days, and ICU-Free Days, in HL and Non-HL Subgroups(Excluding cases with glucose<3.6mmolL)**

| **Outcomes** | **non-HL**  **(n=191)** | | | |  | **HL**  **(n=137)** | | | | †***p*-interaction** |
| --- | --- | --- | --- | --- | --- | --- | --- | --- | --- | --- |
|  | ***β*** | ***SE*** | ***t*** | ***p*** |  | ***β*** | ***SE*** | ***t*** | ***p*** |  |
| 28-day ventilator free days | -0.04 | 1.31 | -0.63 | 0.529 |  | -0.23 | 1.95 | -2.83 | 0.005 | 0.020 |
| 28-day ICU free days | -0.02 | 1.38 | -0.30 | 0.770 |  | -0.21 | 1.64 | -2.67 | 0.009 | 0.029 |
